# Single-Port Transumbilical Laparoscopic Appendectomy: A Preliminary Multicentric Comparative Study in 87 Patients with Acute Appendicitis

**DOI:** 10.1155/2012/492409

**Published:** 2012-05-13

**Authors:** Ramon Vilallonga, Umut Barbaros, Ahmed Nada, Aziz Sümer, Tuğrul Demirel, José Manuel Fort, Oscar González, Manuel Armengol

**Affiliations:** ^1^General Surgery Department, Universitary Hospital Vall d'Hebron, Barcelona 08035, Spain; ^2^General Surgery Department, Istanbul University, Istanbul Faculty of Medicine, Istanbul 34104, Turkey; ^3^General Surgery Department, Cairo University, Cairo 11471, Egypt

## Abstract

*Introduction*. Laparoscopic appendectomy (LA) has been performed in many approaches such as open, laparoscopic and recently Single Port Access (SPAA). In order to elucidate its potential advantages, we compared the two laparoscopic approaches. *Methods*. 87 patients were included in a multicentric study for suspected appendicitis in order to perform (SPAA) appendectomy or laparoscopic appendectomy (LA). All outcomes, including blood loss, operative time, complications, and length of stay and pain were recorded prospectively. *Results*. There were 46 patients in the SPAA group and 41 in the LAG with a mean operative time of 40,4 minutes in the SPAA group and 35,0 minutes in the LA group. Only one patient was converted to an open approach. We described only 2 complications. Pain was graded 2,8 in the SPAA group and 2,9 in the LA group, according to the AVS after 24 hours. Patients in the SPAA Group were more satisfied (7,5 versus 6,9) (*P* < 0.05). Same results were found for the cosmetic result (8,6 versus 7,4) (*P* < 0.05). *Conclusion*. Using the single port approach feasible and safe. The true benefit of the technique should be assessed by new randomised controlled trials.

## 1. Introduction

Nowadays, minimally invasive surgery has increased in its use [1]. A new era has been opened with recent innovations that have pioneered the use of single-incision laparoscopic surgery (SILS) or Single Port Access (SPA). This novel technique or approach may be placed between the pure NOTES surgery, the hybrid NOTES surgery, and the standard laparoscopic surgery [2–5]. Appendectomy is the most common abdominal emergency operation performed in the western world. Some reasons have made that more and more appendectomies are currently performed laparoscopically such as advantages to patients in terms of more accurate diagnosis, diminished wound infections, possibility to treat obese patients, and a more rapid recovery [6]. First report of single-puncture laparoscopic appendectomy technique was performed in 1992 and showed the new approach as a safe, inexpensive, and effective alternative to the currently used multiple-puncture method [7]. 

The new transumbilical approach seems to reduce the trauma of surgical access with its improvement of the postoperative pain and patient cosmesis compared to standard laparoscopic approach. However, other important issues must be critically analysed such as time consumed complications, and difficulties to perform this novel technique. This new technique has been introduced to the surgical community, and we have concentrated on knowing about the feasibility, safety, and clinical advantage of the method. For these reasons, in order to implement SPA appendectomy (SPAA), and know its difficulties, limitations, or advantages, we conducted this multicentre study. The aim of the study is to know if SPA would offer similar operative time, length of stay, and complication profile with improved cosmesis and less postoperative pain in comparison to traditional multi-incision laparoscopic appendectomy or also called standard laparoscopic appendectomy (LA).

## 2. Patients and Methods

In this study, 92 patients (Table 1) underwent SPA appendectomy and standard laparoscopic appendectomy. Three different teams of surgeons in three different hospitals performed the interventions: Vall d'Hebron Hospital (Barcelona, Spain), Cairo University Hospital (Cairo, Egypt), and Istanbul Faculty of Medicine (Istanbul, Turkey). All the three surgeons were trained expert surgeons in laparoscopy and had already performed SILS cholecystectomy previously. All the patients were informed about the intervention technique and provided written informed consent. All the patients had a suggestive clinical diagnosis of acute appendicitis. All patients included in the study were from patients undergoing urgent surgery. Each patient in each hospital was included alternatively in each treatment group (SPAA group and LA group).

### 2.1. Operative Technique

The two surgical techniques were established in both the study and control groups according to a consensus approved by the authors previous to the beginning of the study and according to the different hospital possibilities. Patients were divided into two different groups: SPAA group (SPAAG) and LA group (LAG). For the SPAAG, a single intraumbilical 22 mm incision was made, and the umbilicus was pulled out, exposing the fascia in SPAAG. The surgeons in this study completely extroflexed the umbilicus and a skin incision was made longitudinally for about 1,5 to 2 cm. Two types of trocars were used in the SPAAG and that were currently manufactured for this purpose: the TriPort (Advanced Surgical Concepts, Wicklow, Ireland) and the SILS Port (Covidien, Inc., Norwalk, CT, USA). For the patients included in the LAG, standard trocars were used. All trocars were placed under direct vision. Pneumoperitoneum was maintained at 14 mmHg with carbon dioxide (CO_2_). The abdominal cavity was explored with a 10 mm 30° standard scope in both groups. The patients were then put in a Trendelenburg position and rotated to the left.

In some patients in the SPAAG, reticulating instruments were used to create the necessary operative angle, according to technical difficulties (Reticulating Endo Mini-Shears; Autosuture and Reticulating Endograsp, 5 mm; Autosuture).

The appendicular artery was first exposed, and then clipped if necessary with a standard 5 mm clip applier or cauterized by bipolar grasper.

Two endoloops were used at the stump of the appendix and then divided.

Then, in both groups, a 5 mm 30° standard scope was used in order to extract the specimen. Careful control of homeostasis was then achieved, and drainage was left in place according to surgeon's personal criteria. The fascial incisions were closed with an absorbable suture, and the umbilicus was restored with absorbable cutaneous stitches to its anatomic position. The rest of skin incisions were closed with absorbable cutaneous stitches.

Intraoperative complications such as bleeding, drain placement, surgical times (trocar(s) placement, and surgical dissection and closure) were calculated. The uniformity of anaesthetic technique could not be established because of the different teams involved in each case. Postoperative complications and time for discharge have also been analysed. Pain referred by patients after 12 hours was measured with VAS [8]. All patients received paracetamol 1 g/8 h i.v. as a standard analgesic treatment. During the followup in the outpatient clinic, other data such as hernia or other complications were evaluated. The patients in the outpatient clinic, at one month after surgery, answered two questions: “*How much satisfied with the surgery are you? (0–10)*” and “*How satisfied are you with the cosmetic result of the surgery? (1–10)*.” These short questions pretended to know about the degree of satisfaction and the satisfaction with the cosmetic result.

### 2.2. Statistical Analysis

Treatments for acute appendicitis, LA versus SPAA, were compared using *t*-test for continuous variables (age, times, bleeding, oral intake, discharge, pain at 12 hours, degree of satisfaction, and satisfaction of cosmetic result) and Pearson's chi-square test for categorical variables (sex, appendix dissection and section, complications, and result pathology). *P* < 0.05 was considered significant. Analysis was performed using Stata (StataCorp. 2007. Stata Statistical Software: Release 10. College Station, TX: StataCorp LP)

## 3. Results

Between July 2009 and March 2010, 87 patients were randomized for suspected appendicitis into the SPAA group (SPAAG) or an LA group (LAG). There were 46 patients in the SPAA group and 41 in the LA group. The mean age of the patients was 34,2 (17–73) for the SPAA group and 37,7 (19–69) for the LA group. There were 19 males and 27 females in the SPAA group and 22 males and 19 females in the LA group (Table 1).

SILS Port was used in 38 patients and TriPort in 8 patients and there was no technical difference between them.

In spite of technical difficulties and disorientation specially in the first few cases, the mean operative time was 40,4 minutes in the SPAA group and 35, 0 minutes in the LA group (*P* = 0,110).

In only 1 patient of the SPAA group, the procedure was converted to an open approach due to technical difficulties in a colonic cancer diagnosed during the surgery. Complications occurred in 2 patients, all in the SPPA group. First patient presented with acute coronary syndrome during the surgery; another young woman suffered of an acute pulmonary oedema caused by an allergic reaction to Dexketoprofen who required 5 days endotracheal intubation. The two patients presented a long hospital stay (7 days, 11 days, and 10 days resp.). All these hospital stays have been included in the mean of the postoperative stay at hospital. Drains have been used in 8 and 5 patients in each group because of the local peritonitis found (Tables 2 and 3).

Oral intake was accomplished after 12,5 hours in the SPAA group and 10,7 hours in the LA group. The mean hospital stay was 44,4 hours in the SPAA group (mean 14–264) and 34,0 hours (mean 11–96) in the LA group. Pain was evaluated and was 2,8 in the SPAA group and 2,9 in the LA group, according to the AVS after 24 hours. The degree of satisfaction was higher in the SPAA group (7,5 versus 6,9) (*P* < 0.05). Same results were found for the cosmetic result (8,6 versus 7,4) (*P* < 0.05). At three-month followup, no hernia or other complications have appeared.

## 4. Discussion

Many surgical research groups have developed new surgical technique called Natural Orifice Transluminal Endoscopic Surgery (NOTES) [9]. Some appendectomies have even been performed through a vaginal approach, without visible scars [10]. However, many authors consider that umbilicus a natural orifice since its origin. For this reason, many authors have reported the feasibility of LA with a transumbilical approach, especially in children [11]. Also, some studies investigated the feasibility of SPAA in study populations ranging from 1 to 200 patients, and there is not a standard use of size port in the LAG [12]. As most surgeons, we used conventional ports with a variety of different-sized instruments.

Also, the umbilical access is a well-known and standardized site for access to the abdominal cavity for laparoscopic procedures [13]. However, many authors have described an SPA appendectomy as a step toward less invasive surgical procedures [14]. According to surgeon's experience, umbilical access does not add new risks, and it makes the operating view the same as in standard laparoscopic appendectomy. In this study no differences were found comparing the trocar placement time of each group, and all the trocars were placed under direct vision.

Once the pneumoperitoneum is performed, both techniques can allow making an intraoperative differential diagnosis with other pathologies [15]. In our series, examination of distal ileum, female genital organs including the tubes and the ovary, and other organs situated in pelvic area can be accomplished without difficulties. We had to reconvert to an open surgery approach in a cecal carcinoma misdiagnosed preoperatively.

When the fascia is exposed, it is possible to enter the abdominal cavity with various devices such as 10 mm trocar and two 5 mm trocars. The single-port technique allows easy use of a 10-mm instrument if needed without the burden of having to work with a 5 mm and a 10 mm port so close together.

Due to the vicinity of the ports at the fascial plane in the umbilicus, the operative technique can be more difficult. In some cases the crossing of the instruments (or specially designed instruments) makes the procedure more challenging and initiating new learning curve for surgeon. It has not been defined yet the number of cases needed to gain good experience in SPAA. But it seems that 10 cases should be the number in order to perform a correct learning curve with previous experience in laparoscopic surgery [16].

In our opinion appendectomy is relatively easy operation performed in a relatively safe abdominal area (no much vital organs). This novel approach should probably be the first one to be considered before beginning SPA cholecystectomies, which are more demanding.

When drain is required, right side placement is suitable and can be placed under direct vision.

A very important issue is to consider the conversion from single-incision (SPAA) technique to standard laparoscopic technique. Fear from intraoperative complications is due to inadequate visualization or mobilization of the appendix. For this reason, we consider that a two-port or three-port conversion should not be considered a failure or complication. This concept is very important and is absolutely mandatory in emergency surgeries. An optimum safe view must be achieved. If this is not achieved then the addition of ports is recommended. The opinion of the authors concerning the visualization in this series was not as optimal as with typical laparoscopy. However, a recent report shows that the suprapubic trocar placement shows better benefits in case of retrocecal or purulent or gangrenous acute appendicitis. Trocar placement via the suprapubic approach makes access to and dissection of the appendix easy, and it also enables exteriorization of a drain without adding new lateral incisions [17].

When the diagnosis was established, we found the appendix oedematous, gangrenous, perforated with varying degree of peritonitis, or even associated with peritoneal abscess. According to our short limited experience, we think SPAA technique seems to be suitable for the variety of appendicitis.

Because of the initial experience and the cosmetic research, SPAA has been performed in nonobese and obese patients. According to the literature especially obese patients benefit from LA compared to open one [6, 18]. Unfortunately, at the time of the randomization, the BMI was not calculated but retrospectively analysed, the BMI of the SPAAG is not different from LAG. This is probably because of the lack of experience in the first cases, the fear of umbilical closure, and the search of a better cosmetic result in young women. Many of our patients were adolescent females who may be very aware of their body image.

It seems reasonable to think that the benefits of transition from standard laparoscopic approach to SPAA will be easier than the transition from open to laparoscopic appendectomy.

Accordingly, we believe that the use of this approach for appendectomy is worthwhile. SPAA can be performed properly by one straight instrument and one curved instrument, and even by two standard straight instruments, making the procedure easier compared to use of two curved instruments. New devices and new technology is now available at the time of writing that makes this technique easier.

Concerning the cosmetic result, at the end of the procedure, surgeons took time performing a careful reconstruction of the umbilicus in both groups. Cosmetic results show that there is a certain advantage of performing the single-incision surgery compared to standard one. Patients seem to be more satisfied with the overall result and with the cosmetic result. However, this is a difficult subjective opinion and difficult to measure. According to other authors, the issue of the influence of abdominal scar on the cosmetic and body image showed no difference between open and traditional laparoscopic appendectomies [19]. Our patients are more satisfied with the SPAA than LA (*P* < 0,05), but the importance of abdominal scar may be age and sex related. There is a feeling that young nurses would have scarless operation rather than LA or even open approach. Some authors suggest that suprapubic SILS appendectomy offers better, cosmetically appealing results than the standard umbilical access [17]. However, the data generated by the use of our questionnaire is of dubious quality and cannot be used to make any meaningful statements on satisfaction and cosmetics because it has not been validated.

Recent technologic development has enabled the wider acceptance of new approaches in laparoscopic surgery such as SPAA. All recent data show that the technique is feasible, safe, but will require new randomized studies in order to clarify its indications and a cost effectiveness study of this novel technique will seriously be required [20].

## 5. Conclusion

Single-incision laparoscopic surgery is a feasible way to perform appendectomy. This includes obese patients, uncomplicated and complicated appendicitis as well as exploratory laparoscopy. Conversion to a three-port operation should be done in any case when optimal or suboptimal conditions are not present. As patients' safety was the most important patients with acute appendicitis should be the ones in order to begin the SPAA technique.

The expense and added operative time should be evaluated if it provides the patients with minimal, if any, apparent scarring. Patients are more satisfied with SPAA than LA approach regarding the cosmetic result.

 Refinements in instrumentation will enable wider use of this novel minimally invasive approach. The true benefit of the technique should be assessed by new randomised controlled trials.

## Figures and Tables

**Table 1:** Demographic data of the Single Port Access Appendectomy Group (SPAA Group) and the Laparoscopic Appendectomy Group (LA Group).

	SPAA group (SPAAG) *N* = 46	Laparoscopic appendectomy group (LAG) *N* = 41	*P* value
Age (years), mean (sd)	34,2 (13,3)	37,7 (13,2)	0,227
Gender, *n* (%)			0,287
Male	19 (41,3)	22 (53,7)	
Female	27 (58,7)	19 (46,3)	

**Table 2:** Results concerning operative technique.

	SPAA group (SPAAG) *N* = 46	Laparoscopic appendectomy group (LAG) *N* = 41	*P* value
*Trocar use *			
(i) Covidien SILS	39	—	
(ii) Olympus TriPort	8	—	
Time of trocar(s) introduction (minutes), mean (SD)	5,9 (3,0)	5,8 (1,4)	0,788
Time of surgical dissection (minutes), mean (SD)	40,4 (17,5)	35,0 (13,6)	0,110
Time of closure (minutes), mean (SD)	6,5 (2, 3)	5,6 (1,3)	0,027
Conversion to laparoscopic or open	1 (colonic cancer)	0	0,342
Bleeding (mL), mean (SD)	7 (15,2)	5 (12,2)	0,763
Drainage, *n* (%)	4 (12,2)	5 (8,7)	0,729

**Table 3:** Postoperative results and outcomes.

	SPAA group (SPAAG) *N* = 46	Laparoscopic appendectomy group (LAG) *N* = 41	*P* value
Oral intake (after hours), mean (SD)	12,5 (20)	10,7 (21)	0,962
Discharge (hours), mean (SD)	44,4 (51)	34,0 (20)	0,225
*Complications *			
(i) One patient had acute coronary syndrome during the surgery; another had acute pulmonary oedema	2	0	0,178
(ii) Seroma	0	0	—
(iii) Hernia	0	0	—
Pain at 12 hours (AVS), mean (SD)	2,8 (0,90)	2,9 (0,78)	0,774
Degree of satisfaction, mean (SD)	7,5 (1,0)	6,9 (1,2)	0,009
Satisfaction of aesthetic result, mean (SD)	8,6 (0,9)	7,4 (1,3)	<0,001
Pathology, *n* (%)			1,000
(i) Acute appendicitis	29 (63)	28 (68)	
(ii) Perforated appendicitis	15 (33)	13 (32)	
(iii) Chronic appendicitis	1 (2)	0	
(iv) Colonic neoplasm	1 (2)	0	
